# A comprehensive multi-domain dataset for mitotic figure detection

**DOI:** 10.1038/s41597-023-02327-4

**Published:** 2023-07-25

**Authors:** Marc Aubreville, Frauke Wilm, Nikolas Stathonikos, Katharina Breininger, Taryn A. Donovan, Samir Jabari, Mitko Veta, Jonathan Ganz, Jonas Ammeling, Paul J. van Diest, Robert Klopfleisch, Christof A. Bertram

**Affiliations:** 1 Technische Hochschule Ingolstadt, Ingolstadt, Germany; 2 Pattern Recognition Lab, Friedrich-Alexander-Universität Erlangen-Nürnberg, Erlangen, Germany; 3 Department Artificial Intelligence in Biomedical Engineering, Friedrich-Alexander-Universität Erlangen-Nürnberg, Erlangen, Germany; 4 Department of Pathology, University Medical Center Utrecht, Utrecht, The Netherlands; 5 Schwarzman Animal Medical Center, New York, USA; 6 Department of Neuropathology, Universitätsklinikum Erlangen, Friedrich-Alexander-Universität Erlangen-Nürnberg, Erlangen, Germany; 7 Medical Image Analysis Group, Eindhoven University of Technology, Eindhoven, the Netherlands; 8 Institute of Veterinary Pathology, Freie Universität Berlin, Berlin, Germany; 9 Institute of Pathology, University of Veterinary Medicine Vienna, Vienna, Austria

**Keywords:** Prognostic markers, Tumour biomarkers, Translational research, Cancer

## Abstract

The prognostic value of mitotic figures in tumor tissue is well-established for many tumor types and automating this task is of high research interest. However, especially deep learning-based methods face performance deterioration in the presence of domain shifts, which may arise from different tumor types, slide preparation and digitization devices. We introduce the MIDOG++ dataset, an extension of the MIDOG 2021 and 2022 challenge datasets. We provide region of interest images from 503 histological specimens of seven different tumor types with variable morphology with in total labels for 11,937 mitotic figures: breast carcinoma, lung carcinoma, lymphosarcoma, neuroendocrine tumor, cutaneous mast cell tumor, cutaneous melanoma, and (sub)cutaneous soft tissue sarcoma. The specimens were processed in several laboratories utilizing diverse scanners. We evaluated the extent of the domain shift by using state-of-the-art approaches, observing notable differences in single-domain training. In a leave-one-domain-out setting, generalizability improved considerably. This mitotic figure dataset is the first that incorporates a wide domain shift based on different tumor types, laboratories, whole slide image scanners, and species.

## Background & Summary

Predicting the biological tumor behavior using histopathology is a central requirement for the identification of therapeutic options and the planning of tailored therapy. For this, micrometer-thin sections of tissue are produced from a formalin-fixed and paraffin-embedded tissue block and subsequently stained with histochemical dyes (e.g., hematoxylin & eosin (H&E)) that highlight morphological patterns of the tumor. Several histological patterns are evaluated in a standardized manner and combined to tumor-type specific grading systems^1,2^. The density of mitotic figures, i.e., dividing cells, is a key component for prognostication and part of many grading systems (including human and canine breast carcinoma^1,2^, human neuroendocrine tumors^3^, human and canine lung adenocarcinoma^1,4^, canine lymphosarcoma^5^, canine mast cell tumor^6^, and human and canine soft tissue sarcoma^1,7^). Usually, the number of mitotic figures in a standardized region of interest (ROI), i.e., the mitotic count (MC), is incorporated into the grade by introducing multiple thresholds, e.g., for low, medium and high mitotic activity^1^. However, the identification of mitotic figures is subject to high intra- and inter-rater variability, resulting in low reproducibility of the MC^8–10^. Besides object-level differences, selection of the ROI with the assumed highest mitotic count in the entire histological section(s), as requested by the guidelines^1,11,12^, is prone to significant inter-rater differences^10^. Consequently, the computerized identification of mitotic figures in digitized whole slide images (WSIs) is a relevant topic of ongoing scientific interest, after previous attempts with classical image analysis using special stains^13^.

Especially since the advent of deep learning, automatized approaches have reached or even exceeded the performance of human experts and have shown a high potential to improve this prognostic task^8,10,14^. The development of deep learning-based algorithms was primarily supported by the availability of open datasets, such as the challenge datasets of the MITOS 2012 and 2014 challenges^15,16^, the AMIDA13 challenge^17^, and the TUPAC16 challenge^18,19^. All of these challenge datasets used human breast carcinoma images and have since been complemented by two datasets covering two canine tumor types (breast carcinoma and mast cell tumors)^20,21^ annotated on the complete WSI. Besides their significant merit in the field of mitosis detection, these existing datasets are mostly limited to single image domains, i.e., they include only a single imaging device (whole slide image scanner), lab environment (tissue sectioning, staining, etc.), species, or tissue/tumor type. The only exception in this regard is the TUPAC16 data set, which included two scanners and three labs. As was recently shown, deep learning methods for mitotic figure detection can severely degrade in the presence of domain shifts^22,23^. This limits the use of deep learning-based mitotic figure detectors for a wide application in tumor research and in clinical workflows.

This motivated the inception and conduction of the 2021 MItosis DOmain Generalization (MIDOG) challenge, held in conjunction with the 25th International Conference on Medical Image Computing and Computer Assisted Intervention (MICCAI 2021). The objective of this challenge was to tackle the domain shift caused by the digitization device on histopathology images of human breast carcinoma. In 2022, the succeeding MIDOG 2022 challenge extended the previous dataset with a specific focus on different tumor types from humans and dogs, while ensuring high domain variability from several imaging devices and lab environments^24^. By combining numerous domains in a single dataset (as opposed to creating multiple smaller datasets from various sources) the dataset benefits from homogeneous selection/inclusion criteria, and a consistent annotation process, that allows for a high comparability of algorithms across domains.

In this work, we present and describe MIDOG++, an extended version of the dataset that was previously made available as the training sets of the MIDOG 2021 and 2022 challenges as well as an extensive evaluation of this dataset. Each image represents a distinct case from seven human or canine tumor types, digitized by one out of five whole slide scanners. The original MIDOG training datasets^25^ provided 354 annotated images across five different cancer types. We extend this dataset by providing images and/or annotations for another 149 cases on two additional tumor types (canine soft tissue sarcoma, human melanoma) (see Table 1). This mitotic figure dataset is the first that provides images from various domains with a particular focus on different tumor types. While there are some datasets that allow testing generalizability of algorithms between single domains, particularly different scanners such as the MIDOG 2021 dataset, this dataset is the first that includes many sources for domains shifts relevant for diagnostic pathology.

**Table 1:** Overview of the domains of the dataset.

Domain	No. Cases	Tumor Type	Origin	Species	Scanner	Resolution	Comment
1a	50	breast carcinoma	UMC Utrecht	human	Hamamatsu XR (C12000-22)	$0.23\frac{\mu m}{px}$	a), b)
1b	50	breast carcinoma	UMC Utrecht	human	Hamamatsu S360 (0.5 N/A)	$0.23\frac{\mu m}{px}$	a), b)
1c	50	breast carcinoma	UMC Utrecht	human	Leica ScanScope CS2	$0.25\frac{\mu m}{px}$	a), b)
2	44	lung carcinoma	VMU Vienna	canine	3DHistech Pannoramic Scan II	$0.25\frac{\mu m}{px}$	b)
3	55	lymphosarcoma	VMU Vienna	canine	3DHistech Pannoramic Scan II	$0.25\frac{\mu m}{px}$	b)
4	50	cutaneous mast cell tumor	FU Berlin	canine	Aperio ScanScope CS2	$0.25\frac{\mu m}{px}$	b)
5	55	neuroendocrine tumor	UMC Utrecht	human	Hamamatsu XR (C12000-22)	$0.23\frac{\mu m}{px}$	b)
6a	85	soft tissue sarcoma	AMC New York	canine	3DHistech Pannoramic Scan II	$0.25\frac{\mu m}{px}$	
6b	15	soft tissue sarcoma	VMU Vienna	canine	3DHistech Pannoramic Scan II	$0.25\frac{\mu m}{px}$	
7	49	melanoma	UMC Utrecht	human	Hamamatsu XR (C12000-22)	$0.23\frac{\mu m}{px}$	c)
total	503						

In total, regions of interest from 503 tumor cases have been included. a) annotations and images were part of MIDOG 2021^30^, b) annotations and images were part of MIDOG 2022^25^, c) images (but no annotations) were part of MIDOG 2022.

## Methods

The following section describes the sample collection and preparation for the specimens included in the presented dataset. Furthermore, we elaborate on data annotation and the methods used for validating the presented dataset and annotation database.

### Specimen preparation and digitization

For this dataset, seven different tumor types (domain 1–7, listed in Table 1), for which the MC has high prognostic relevance (see above), were selected. The cases of three tumor types were obtained from human patients (breast carcinoma, pancreatic and gastrointestinal neuroendocrine tumors, and cutaneous melanoma) and four tumor types from canine patients (pulmonary carcinoma, lymphosarcoma mostly in lymph nodes, cutaneous mast cell tumors, and (sub)cutaneous soft tissue sarcoma). All tumor specimens have been submitted to the respective pathology laboratory for routine diagnostic service and histological sections were either retrieved from the diagnostic archive or produced from archived tissue blocks using the routine processing steps of the laboratory. For the animal tissue, all sections were sent in by veterinary practices and clinics. For the human specimens, institutional review board (IRB) approval was obtained (TCBio 20–776, UMC Utrecht). The approval includes the anonymized publication of the digitized histopathology samples. For the canine cases, no IRB approval was required for the retrospective use of the diagnostic specimens. All histological sections were stained with standard H&E dye and scanned with one out of five scanners (see Table 1) with an objective lens with a magnification of 40× resulting in a scan resolution of either $0.25\frac{{\rm{\mu m}}}{{\rm{px}}}$ or $0.23\frac{{\rm{\mu m}}}{{\rm{px}}}$ (see Table 1). In each case, the standard settings of the respective laboratory were used for scanning.

After digitization, a pathologist (C.A.B.) selected a region of interest within each WSI spanning exactly 2 mm^2^ with an aspect ratio of 4:3. This region of interest was defined as a tumor area with appropriate tissue and scan quality and high mitotic density, according to current guidelines^1,11,26,27^. Cases with particularly poor tissue or scan quality throughout the WSI were excluded from the dataset. The region of interest size of 2 mm^2^ was chosen since it approximates the area of 10 fields at 400× optical magnification of standard light microscopes (high-power filed (HPF)), which is the routine approach^1,11,26,27^, and has therefore been used for this and previous mitotic figure datasets^17,18^. Due to differing scan resolutions between the different scanners, the resulting image size varies mildly between the domains. The original image formats use varying lossy compression settings as in the default settings in the respective manufacturers’ software. The selected region was cropped and exported in the TIFF format using lossless compression for each case.

### Annotation methods

The annotations were created according to previously established standards^14,19,20^. The seven domains were annotated in separate workflows, meaning that all images of one domain were processed at the same time. A pathologist (C.A.B.) screened all images of one domain twice in the H&E stained sections using the screening mode of the software SlideRunner^28^ and annotated each mitotic figure as well as structures with similar morphology (hard negatives) with the respective class label. All structures of interest were marked by a circular annotation with a radius of 50 pixels with the center coordinate in the approximate center of the structures. The hard negative class was solely provided as discriminative annotation toward the mitotic figure class and non-exhaustively annotated with the objective of reaching an object count in the same order of magnitude as the mitotic figure class per tumor type.

Regardless of the rigorous screening by the pathologist, it can be expected that some mitotic figures were overlooked^17,19–21^. To detect these missed candidates, the initial labels from the screening process were used to train a deep learning model (customized RetinaNet as described by Marzahl *et al*.^29^, pre-trained on the MIDOG2021 training dataset for tumor domains 1a-c) using three-fold cross-validation. The model was applied to the images of the respective validation fold to find candidates for mitotic figures that were not part of the initial screening process. This process was performed on each domain independently. Low detection thresholds were used to guarantee a low number of false negative detection results. Another benefit of the low detection threshold was the creation of a high proportion of false positives, which reduced the risk of a confirmation bias for the annotators.

Under the assumption that this rigorous annotation process resulted in a low rate of missed mitotic figures, we then aimed to find a multi-expert consensus. All annotated mitotic figure candidates from the manual annotations and the algorithmic detections were cropped as 128 × 128 pixel-sized patches (png format), which were named according to the label identification number, i.e., blinded to the assigned class label. These patches were sent to a second pathologist (R.K.) who was asked to assign a class label (mitotic figure or hard negative). Labels with an agreement between the two pathologists were directly incorporated in the ground truth database and patches with a disagreed label were sent to a third pathologist (T.A.D) for final decision. This multi-expert label process was conducted to improve the quality of the final labels. The three involved pathologists had a high level of experience with mitotic figure annotation through involvement in diagnostic service and development of previous datasets^19–21^. Classification of mitotic figures against hard negatives was done according to the current guidelines^11,26^.

### Evaluation methods

For technical validation of the dataset, we trained an object detection network for the task of mitotic figure detection. Mitotic figure detection was successfully performed using single- and multi-stage detectors^14^, with no clear advantage for one strategy over the other, and consequently, the simpler, single-stage approach was selected for this evaluation. For this, we stratified a 20% test set of each of the 10 domains summarized in Table 1. We ensured a roughly equal MC distribution among each training and corresponding test subset. In total, this resulted in a hold-out test set covering 111 images/cases.

We first performed a single-domain training, where we trained the object detector on the training subset of each tumor type and evaluated the model across the test sets of all tumor types. To limit the number of experiments and have sufficient support for the respective classes in each domain, we combined subsets a-c of domain 1 and subsets a and b of domain 6, which were the same tumor types, resulting in seven experiments. We used this strategy also to show the potential of the dataset to investigate generalizability across tumor types rather than scanners as these questions may be investigated by focusing on the MIDOG2021 dataset^30^. Afterwards, we conducted a leave-one-out training, where we trained the model on all tumor types but one, and again evaluated the models across the test sets of all tumor types. Finally, we trained the object detector on the complete training set of 392 images/cases. For each experiment, we performed a stratified 5-fold cross-validation (for training and validation set, with the same hold-out test set mentioned previously) and averaged the performance results.

For all experiments, we used the RetinaNet architecture^31^ customized for the task of cell detection on microscopic images^29^. We trained the network with image patches sized 512 × 512 pixels, extracted at the highest magnification level. During each epoch, we sampled 1000 training and 250 validation patches uniformly across all images of the stratified subsets. We followed a guided sampling strategy to account for the rare mitotic figure events: If no mitotic figure was present on the slide, patches were sampled randomly across the 2 *mm*^2^ image. For the remaining slides, 50% of the patches were sampled randomly and 50% of the patches were sampled from a 512-pixel radius around mitotic figure annotations. Hard negatives were disregarded during training, i.e., the classification task of detected objects was designed as a two-class problem (mitotic figure vs. background). The models were trained with a batch size of 12 and a discriminative^32^ learning rate in an interval of [5 × 10^−5^, 5 × 10^−4^]. We trained the models for 200 epochs after which we observed convergence of the validation loss and used the validation set to retrospectively select the model with the highest average precision (AP) for mitotic figure detection. The models were optimized with the standard RetinaNet loss as the sum of the bounding box regression loss (smooth L1 loss) and the instance classification loss (focal loss^31^). During model training, we used the standard augmentations provided by the Fastai v1^33^ framework, including random flipping, affine transformations, and random lightning and contrast change. For each model, the input patches were z-score normalized using the mean and standard deviation of all tissue-containing areas of the respective training images.

For inference on the test set, we used a sliding-window approach with a 10% overlap and removed duplicate detections using non-maximum suppression (NMS). We then evaluated the detected mitotic figure candidates against the ground truth annotations and computed the mean F_1_ score across all test WSIs of each tumor type.

## Data Records

The 2 mm^2^ cropout images are provided on figshare^34^ for public non-restricted access. Annotations are provided in two formats: (1) The annotations for each object together with the class (mitotic figure/non-mitotic figure) of the expert consensus as JSON file, and (2) An SQLite database in the format used by the open source WSI viewer SlideRunner^28^. We extended the MS COCO format to include also the individual labels by each of the experts, which can be found in the labels field of each annotation. Additionally, we provide a *datasets_xvalidation.csv* file, which summarizes the slide-level train/test split used for the results presented in this work in the figshare repository^34^.

The following section provides an overview of the presented dataset including the distribution of mitotic figure and non-mitotic figure annotations across all tumor types included in the database. Furthermore, we elaborate on the inter-annotator concordance for the task of mitotic figure annotation.

### Overall description

**Fig. 1:**
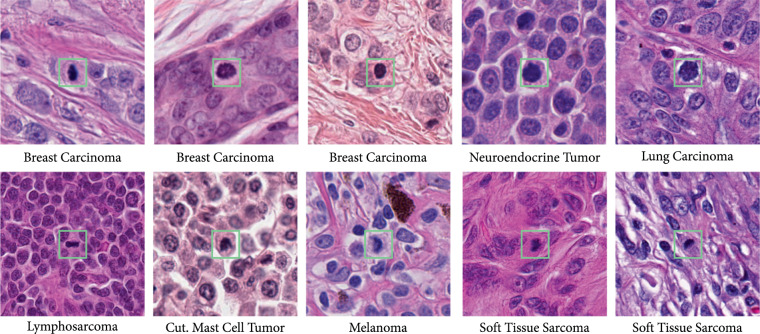
Mitotic figure candidates from all domains summarized in Table 1.

The respective tumor domains show a strong visual representation shift. As seen in Fig. 1, the use of different digitization devices creates a color and depth-of-field variance (see the human breast carcinoma cases). Additionally, the tumor type influences the morphological pattern. For instance, the canine lymphosarcoma tissue showed a considerably smaller average cell size. Similarly, the density of tumor cells varies largely over tumor types. Furthermore, the images of human melanoma contain pigment particles that contribute additional imposter structures to the mitotic figure detection process, although they have a different chromaticity (brown) compared to mitotic figures as shown in Fig. 1.

**Fig. 2:**
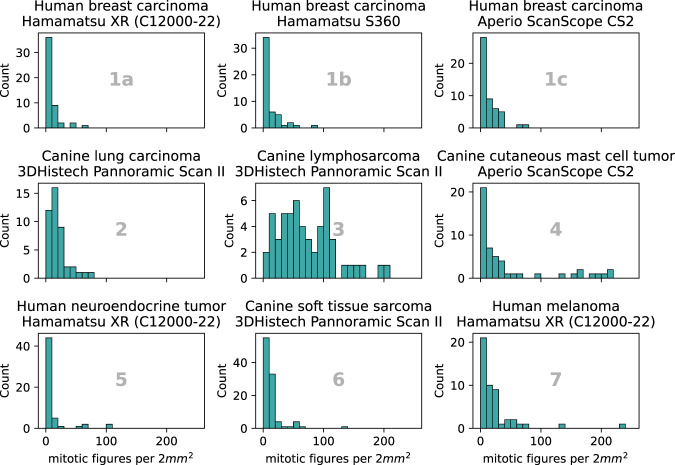
Histogram of the mitotic figures per case per domain. Cases from domains 6a and 6b (canine soft tissue sarcoma) have been aggregated.

The biological differences in tumor morphology are also reflected in the overall MC per 2 mm^2^ area (see Fig. 2 and Table 2). While for the human neuroendocrine tumor, the vast majority of cases only contain very few mitotic figures, the MC for the canine lymphosarcoma is strongly elevated compared to the remainder of tumor types. This is in concordance with expected values for these tumor types and is also reflected by the respective grading scheme by Valli *et al*.^5^, where grades 2 and 3 are distinguished by MCs >60 and >100 per 10 HPFs, respectively. Similarly, the grading system for canine lung adenocarcinoma^35^ comprises four tiers, where the highest grade is represented by an MC exceeding 30 per 10 HPF. In comparison, the current guidelines from the College of American Pathologist^27^ have its highest cutoff value at 15. This is reflected by the lower median MC for human breast carcinoma of 5.5 to 7.5 compared to the median MC of 15.5 for canine lung carcinoma (see Table 2).

**Table 2:** Distribution of mitotic figures and non-mitotic figure (imposter) annotations per scanner and tumor type.

Domain	Tumor Type	Scanner	Cases	Mitotic Figures		Non-Mitotic Figures	
				sum	median	sum	median
1a	breast carcinoma (human)	Hamamatsu XR (C12000-22)	50	451	5.5	724	12.0
1b	breast carcinoma (human)	Hamamatsu S360	50	582	6.0	1,066	14.0
1c	breast carcinoma (human)	Aperio ScanScope CS2	50	688	7.5	924	13.5
2	lung carcinoma (canine)	3DHistech Pannoramic Scan II	44	855	15.5	952	20.5
3	lymphosarcoma (canine)	3DHistech Pannoramic Scan II	55	3,959	66.0	4,257	74.0
4	cutaneous mast cell tumor (canine)	Aperio ScanScope CS2	50	2,327	14.5	1,366	15.0
5	neuroendocrine tumor (human)	Hamamatsu XR (C12000-22)	55	639	4.0	1,762	22.0
6a	soft tissue sarcoma (canine)	3DHistech Pannoramic Scan II	85	1,097	9.0	2,072	17.0
6b	soft tissue sarcoma (canine)	3DHistech Pannoramic Scan II	15	189	7.0	303	14.0
7	melanoma (human)	Hamamatsu XR (C12000-22)	49	1,150	11.0	925	12.0
total			503	11,937		14,351	

### Label agreement

Mitotic figures have notoriously high inter-rater disagreement, which was the reason for our three-expert annotation setup. In previous work by our group, we have shown that an inter-rater count exceeding three does not significantly add to the label stability and the corresponding benefit for machine learning tasks^36^. Assessing the label stability of our two primary experts, we found that in approximately 20% of cases, the experts disagreed in their assignment of mitotic and non-mitotic figures (see Table 3). While the distribution varied across tumor domains, we found that the third expert provided an almost even distribution of mitotic figure and non-mitotic figure labels.

**Table 3:** Distribution of mitotic figures and non-mitotic figures in the three expert rating.

Domain	Two-Expert Rating			Third Expert		Final Distribution	
	mitotic figures	non-mitotic figures	disagreed	mitotic figures	non-mitotic figures	mitotic figures	non-mitotic figures
1a	28.85%	48.00%	23.15%	41.18%	58.82%	38.38%	61.62%
1b	24.21%	57.46%	18.33%	60.60%	39.40%	35.32%	64.68%
1c	28.35%	49.88%	21.77%	65.81%	34.19%	42.68%	57.32%
2	32.17%	44.91%	22.92%	66.18%	33.82%	47.34%	52.66%
3	37.41%	41.64%	20.95%	51.42%	48.58%	48.19%	51.81%
4	49.50%	31.19%	19.31%	69.99%	30.01%	63.01%	36.99%
5	17.38%	61.17%	21.46%	43.11%	56.89%	26.62%	73.38%
6 (a + b)	31.49%	51.90%	16.61%	21.88%	78.12%	35.13%	64.87%
7	44.87%	37.93%	17.20%	61.34%	38.66%	55.42%	44.58%
total	34.92%	45.10%	19.98%	52.50%	47.50%	45.41%	54.59%

Third expert only rated objects that the first two experts disagreed upon.

## Technical Validation

The following section summarizes the performance results of our single-domain and leave-one-out experiments. We report our results as average F_1_ score of the 5-fold cross-validation. Detailed results including the standard deviation across all folds can be obtained from Tables 4, 6. The operating point for each model was optimized on the respective validation split. Tables 5, 7 summarize the AP of the individual models and are thereby independent of the respective operating points. In the following, we address the individual domains by their tumor type, which is expected to be the major source of domain shift (particularly in the leave-one-out experiments). However, we acknowledge that the different tumor type domains include further sources of domain shift (species, laboratory and scanner), which are difficult to separate/group further for detailed experiments.

**Table 4:** Mean and standard deviation of F_1_ score of 5-fold cross-validation for single domain training.

	breast carcinoma (human)	lung carcinoma (canine)	lymphosarcoma (canine)	cutaneous mast cell tumor (canine)	neuroendocrine tumor (human)	soft tissue sarcoma (canine)	melanoma (human)
breast carcinoma (human)	0.71 ± 0.01	0.35 ± 0.16	0.26 ± 0.11	0.67 ± 0.05	0.57 ± 0.06	0.48 ± 0.12	0.78 ± 0.03
lung carcinoma (canine)	0.51 ± 0.02	0.66 ± 0.02	0.55 ± 0.06	0.38 ± 0.07	0.43 ± 0.05	0.62 ± 0.04	0.69 ± 0.03
lymphosarcoma (canine)	0.40 ± 0.06	0.54 ± 0.03	0.79 ± 0.01	0.64 ± 0.03	0.28 ± 0.09	0.42 ± 0.05	0.45 ± 0.07
cutaneous mast cell tumor (canine)	0.52 ± 0.03	0.30 ± 0.07	0.30 ± 0.04	0.85 ± 0.00	0.42 ± 0.09	0.49 ± 0.01	0.62 ± 0.08
neuroendocrine tumor (human)	0.47 ± 0.09	0.40 ± 0.07	0.36 ± 0.13	0.38 ± 0.13	0.58 ± 0.03	0.43 ± 0.07	0.73 ± 0.03
soft tissue sarcoma (canine)	0.60 ± 0.04	0.56 ± 0.05	0.49 ± 0.04	0.58 ± 0.09	0.47 ± 0.05	0.70 ± 0.02	0.63 ± 0.07
melanoma (human)	0.55 ± 0.07	0.37 ± 0.14	0.17 ± 0.09	0.52 ± 0.08	0.59 ± 0.01	0.57 ± 0.04	0.82 ± 0.01
all	0.71 ± 0.02	0.68 ± 0.02	0.73 ± 0.01	0.82 ± 0.01	0.59 ± 0.01	0.69 ± 0.01	0.81 ± 0.01

**Table 5:** Mean and standard deviation of average precision (AP) of 5-fold cross-validation for single-domain training.

	breast carcinoma (human)	lung carcinoma (canine)	lymphosarcoma (canine)	cutaneous mast cell tumor (canine)	neuroendocrine tumor (human)	soft tissue sarcoma (canine)	melanoma (human)
breast carcinoma (human)	0.70 ± 0.03	0.31 ± 0.09	0.22 ± 0.06	0.64 ± 0.09	0.53 ± 0.05	0.43 ± 0.09	0.75 ± 0.05
lung carcinoma (canine)	0.45 ± 0.02	0.62 ± 0.02	0.46 ± 0.06	0.31 ± 0.06	0.53 ± 0.05	0.59 ± 0.03	0.77 ± 0.01
lymphosarcoma (canine)	0.44 ± 0.05	0.50 ± 0.05	0.80 ± 0.01	0.67 ± 0.03	0.55 ± 0.01	0.48 ± 0.05	0.59 ± 0.05
cutaneous mast cell tumor (canine)	0.45 ± 0.02	0.23 ± 0.09	0.23 ± 0.08	0.87 ± 0.02	0.50 ± 0.06	0.40 ± 0.06	0.70 ± 0.01
neuroendocrine tumor (human)	0.40 ± 0.08	0.34 ± 0.07	0.28 ± 0.10	0.32 ± 0.11	0.52 ± 0.03	0.40 ± 0.02	0.67 ± 0.03
soft tissue sarcoma (canine)	0.55 ± 0.05	0.48 ± 0.05	0.41 ± 0.05	0.54 ± 0.11	0.38 ± 0.09	0.69 ± 0.04	0.60 ± 0.09
melanoma (human)	0.46 ± 0.09	0.30 ± 0.10	0.14 ± 0.05	0.46 ± 0.11	0.55 ± 0.03	0.52 ± 0.02	0.80 ± 0.01
all	0.74 ± 0.02	0.65 ± 0.06	0.69 ± 0.04	0.82 ± 0.01	0.62 ± 0.03	0.69 ± 0.03	0.82 ± 0.03

**Table 6:** Mean and standard deviation of F_1_ score of 5-fold cross-validation for leave-one-out training.

	breast carcinoma (human)	lung carcinoma (canine)	lymphosarcoma (canine)	cutaneous mast cell tumor (canine)	neuroendocrine tumor (human)	soft tissue sarcoma (canine)	melanoma (human)
w/o breast carcinoma (human)	0.66 ± 0.01	0.69 ± 0.02	0.74 ± 0.01	0.81 ± 0.01	0.58 ± 0.03	0.68 ± 0.02	0.79 ± 0.01
w/o lung carcinoma (canine)	0.71 ± 0.00	0.63 ± 0.02	0.74 ± 0.01	0.81 ± 0.01	0.60 ± 0.02	0.67 ± 0.02	0.81 ± 0.01
w/o lymphosarcoma (canine)	0.71 ± 0.01	0.64 ± 0.02	0.57 ± 0.03	0.80 ± 0.01	0.58 ± 0.04	0.68 ± 0.02	0.81 ± 0.02
w/o cutaneous mast cell tumor (canine)	0.72 ± 0.01	0.66 ± 0.01	0.74 ± 0.03	0.77 ± 0.02	0.58 ± 0.02	0.69 ± 0.02	0.80 ± 0.01
w/o neuroendocrine tumor (human)	0.72 ± 0.02	0.66 ± 0.03	0.73 ± 0.01	0.82 ± 0.00	0.59 ± 0.03	0.69 ± 0.01	0.81 ± 0.01
w/o soft tissue sarcoma (canine)	0.70 ± 0.01	0.67 ± 0.01	0.74 ± 0.01	0.81 ± 0.01	0.57 ± 0.03	0.65 ± 0.01	0.80 ± 0.01
w/o melanoma (human)	0.70 ± 0.02	0.66 ± 0.01	0.73 ± 0.01	0.82 ± 0.01	0.59 ± 0.03	0.67 ± 0.01	0.79 ± 0.01

**Table 7:** Mean and standard deviation of average precision (AP) of 5-fold cross-validation for leave-one-out training.

	breast carcinoma (human)	lung carcinoma (canine)	lymphosarcoma (canine)	cutaneous mast cell tumor (canine)	neuroendocrine tumor (human)	soft tissue sarcoma (canine)	melanoma (human)
w/o breast carcinoma (human)	0.70 ± 0.02	0.66 ± 0.03	0.72 ± 0.02	0.82 ± 0.02	0.61 ± 0.02	0.67 ± 0.05	0.80 ± 0.02
w/o lung carcinoma (canine)	0.71 ± 0.05	0.65 ± 0.05	0.67 ± 0.08	0.81 ± 0.05	0.61 ± 0.04	0.70 ± 0.02	0.79 ± 0.04
w/o lymphosarcoma (canine)	0.71 ± 0.04	0.64 ± 0.03	0.71 ± 0.03	0.81 ± 0.04	0.60 ± 0.02	0.68 ± 0.02	0.80 ± 0.02
w/o cutaneous mast cell tumor (canine)	0.72 ± 0.02	0.64 ± 0.03	0.65 ± 0.12	0.79 ± 0.06	0.62 ± 0.01	0.69 ± 0.01	0.82 ± 0.02
w/o neuroendocrine tumor (human)	0.73 ± 0.03	0.67 ± 0.02	0.73 ± 0.02	0.83 ± 0.01	0.63 ± 0.02	0.69 ± 0.03	0.81 ± 0.01
w/o soft tissue sarcoma (canine)	0.71 ± 0.01	0.63 ± 0.04	0.70 ± 0.04	0.79 ± 0.06	0.61 ± 0.03	0.68 ± 0.02	0.78 ± 0.02
w/o melanoma (human)	0.71 ± 0.04	0.59 ± 0.08	0.60 ± 0.16	0.77 ± 0.05	0.60 ± 0.03	0.67 ± 0.04	0.79 ± 0.03

### Single-domain training

**Fig. 3:**
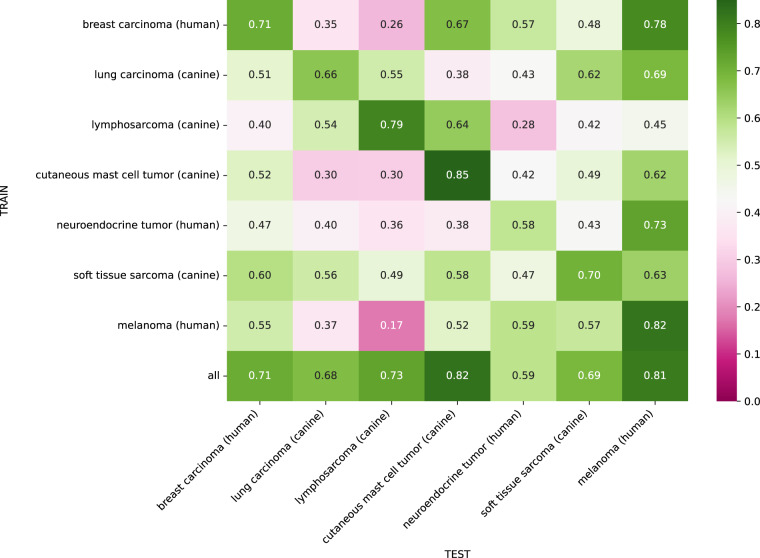
Domain matrix for single domain training. Matrix entry *m*_*i, j*_ is the mean mitotic figure F_1_ score of the 5-fold cross-validation when training on the tumor type in row *i* and testing on tumor type in column *j*. Diagonal elements indicate in-domain performance, whereas off-diagonal elements represent cross-domain performance. The last row summarizes the F_1_ score when training on the training sets of all domains.

Figure 3 summarizes the mean F_1_ score of the 5-fold cross-validation when training on a single tumor type and testing the model across all domains. The in-domain performance on the diagonal of the domain matrix showed considerable performance differences for the different tumor types. The canine mast cell tumor model showed the highest in-domain performance with an F_1_ score of 0.85, closely followed by the human melanoma model with an F_1_ score of 0.82. The human neuroendocrine tumor model achieved the lowest performance with an in-domain F_1_ score of 0.58. The dataset statistics in Fig. 2 and Table 2 show a high number of low-density cases for the human neuroendocrine tumor domain, resulting in a small annotation pool, which is likely to have a negative impact on robust model training. However, testing on the human neuroendocrine tumor shows that no model was able to score an F_1_ score higher than 0.59 on this tumor domain, indicating that mitotic figure detection (by the dataset annotators and/or algorithm) was a difficult task for this domain in general. As shown in Tables 5, 7, the low F_1_ score value also did not originate from a suboptimal threshold setting, but stems from a general low recognition performance on the domain. The off-diagonal elements of the domain matrix in Fig. 3 summarize the cross-domain performance of the single-domain models. Generally, the models show a considerable decrease in cross-domain F_1_ score compared to in-domain, which highlights the inherent domain shift of the presented dataset. The visualization shows that some models, e.g. the model trained on the canine soft tissue sarcoma domain, generalize comparably well, while other models, e.g., the model trained on the canine cutaneous mast cell tumor domain, encounter difficulties for many tumor type domains. Interestingly, for the human neuroendocrine tumor domain the model trained on human melanoma domain slightly outperformed the in-domain model. This, again, could be explained by the low total number of mitotic figures seen when training on the human neuroendocrine tumor samples, which might have hindered robust training. The human melanoma column of the domain matrix in Fig. 3 shows comparably high performance for all models on this domain. Typically, melanomas can have very mixed morphological growth patterns, resembling many of the tumors included in the presented dataset, which might have eased generalization to melanoma for models trained on other tumor types. Furthermore, the results in Table 3 show that human melanoma was one of the tumor types with the lowest inter-rater variability, indicating that mitotic figure detection was less difficult for this tumor type in general.

Of note, four out of six models scored worst on lymphosarcoma in the cross-domain performance (see Table 4). This indicates a large domain gap, which is in line with the perceptual difference caused by smaller average cell sizes (Fig. 1).

### Leave-one-domain-out training

**Fig. 4:**
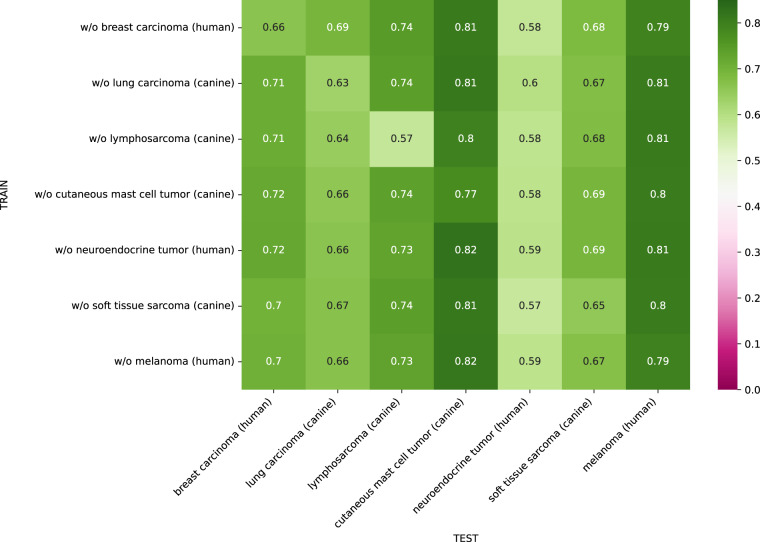
Domain matrix for leave-one-out training. Matrix entry *m*_*i,j*_ is the mean mitotic figure F_1_ score of the 5-fold cross-validation when training without the tumor type in row *i* and testing on tumor type in column *j*. Diagonal elements indicate out-of-domain performance, whereas off-diagonal elements represent in-domain performance.

Figure 4 shows the domain matrix for the leave-one-out training, where each model has been trained on all tumor type domains but one. Overall, the results show that increasing the variability of the training subset by including a higher number of domains but also of cases improved the model performance on the test sets. For each tumor type column, the performance scores are fairly consistent over the different training set compositions. The results again highlight that the human neuroendocrine tumor domain was the most difficult domain for the models while the human melanoma and canine mast cell tumor domain produced the highest F_1_ score of 0.81. In case of the neuroendocrine tumor, the drop in performance is likely caused by class imbalance and the low count of mitotic figures in this dataset (see Fig. 2), making the object detection problem significantly more challenging.

The canine lymphosarcoma domain shows the strongest domain shift, visible from both a weak generalization to other domains when trained on lymphosarcoma and the worst performance in the leave-one-domain-out generalization assessment.

### Evaluation on the MIDOG 2022 test set

To test the domain generalization of the trained models, we applied them to the test set of the MIDOG 2022 challenge^24^. The test set covered 100 ROIs equally distributed across ten tumor types: human melanoma, human astrocytoma, human bladder carcinoma, canine mammary carcinoma, canine mast cell tumor, human meningioma, human colon carcinoma, canine hemangiosarcoma, feline soft tissue sarcoma, and feline lymphosarcoma. The human melanoma and canine mast cell tumor samples were disjoint (i.e., had a different domain) from the samples included in the dataset presented in this work. In particular, the canine cutaneous mast cell tumor cases were from a different lab (VMU Vienna) and scanned with a different scanner (3DHistech Pannoramic Scan II), and the melanoma cases were digitized using a different scanner (3DHistech Pannoramic Scan II).

**Fig. 5:**
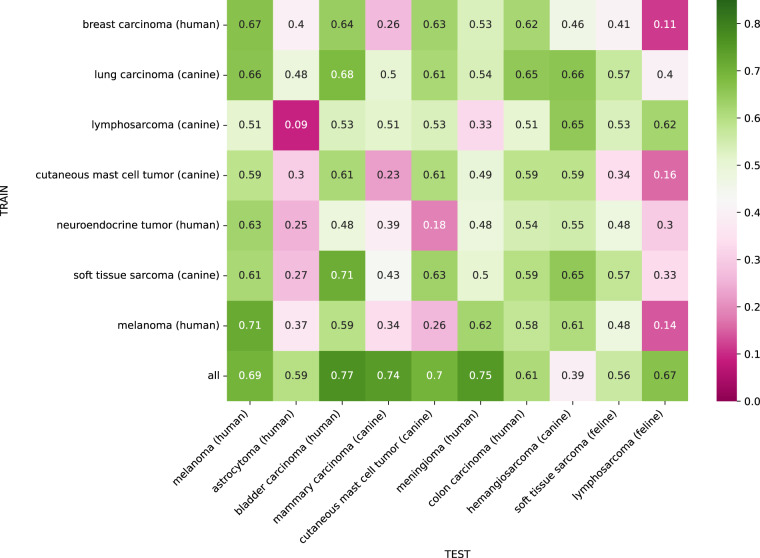
Domain matrix for single domain training when deploying the models to the unseen test set of the MIDOG 2022 challenge. Matrix entry *m*_*i,j*_ is the mean mitotic figure F_1_ score of the 5-fold cross-validation when training on the tumor type in row *i* and testing on tumor type in column *j*.

The domain matrix in Fig. 5 summarizes the cross-domain performance of our single-domain models on the MIDOG 2022 test set. Generally, the single-domain models show low generalization across most tumor types with the highest F_1_ score of 0.71 achieved by the canine soft tissue sarcoma model when being applied to human bladder carcinoma. The most difficult tumor doamin was the human astrocytoma, where no single-domain model achieved an F_1_ score higher than 0.48 (e.g., the canine lymphosarcoma model completely failed with an F_1_ score of 0.09). As with the neuroendocrine tumor, we expect this to be explained by class imbalance generally lower MC counts for this domain.

The feline lymphosarcoma was also a challenging domain for most single-domain models except for the canine lymphosarcoma model, which achieved an F_1_ score of 0.62. This further highlights the particular domain shift between lymphosarcoma and other solid tumors of our evaluation in their visual representation, as also immanent from the smaller average cell size. The last row of the domain matrix in Fig. 5 summarizes the F_1_ score for the model trained on all seven tumor types, which shows a comparably good generalization performance across all tumor types except for canine hemangiosarcoma. This validates the general assumption that high variability of training data increases the domain generalization capability of neural networks.

**Fig. 6:**
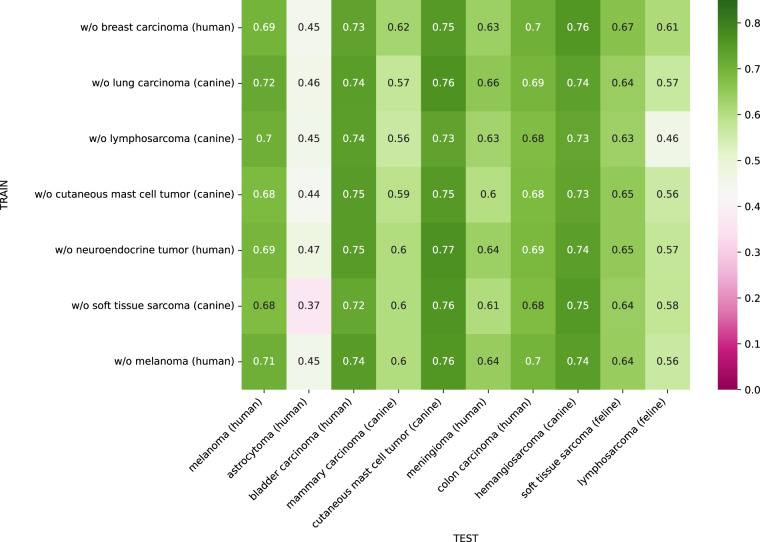
Domain matrix for leave-one-out training when deploying the models to the unseen test set of the MIDOG 2022 challenge. Matrix entry *m*_*i,j*_ is the mean mitotic figure F_1_ score of the 5-fold cross-validation when training without the tumor type in row *i* and testing on tumor type in column *j*.

Figure 6 summarizes the cross-domain F_1_ score for the leave-one-out models when being applied to the MIDOG 2022 test set. The results show that the models trained on six domains overall show a good generalization across the unseen tumor types except for human astrocytoma, where all models faced difficulties and scored a maximum F_1_ score of 0.47. Furthermore, the results show that when not including canine lymphosarcoma in the training database, the model performance considerably declined on the feline lymphosarcoma, which could again be explained by the varying average cell size of lymphosarcomas. Finally, the results indicate that the domain shift between different animal species may be negligible for the task of mitotic figure detection as the models did not show a significant performance drop on feline tumor types compared to human and canine tumor types, which constituted the training database.

### Dataset insights from algorithm development

Overall, the results presented in the technical validation highlight the domain shift inherent in the multi-domain dataset for mitotic figure detection presented in this work. Furthermore, they show that neural networks can achieve a certain level of domain generalization if they are trained on a diverse dataset, thus highlighting the need for open-source datasets that cover a wide range of domains. By covering multiple species, tumor types, pathology laboratories, and whole slide scanning systems we intended to cover this domain diversity from as many aspects as possible, which allows for validating the domain generalization capability of developed algorithms in multiple domain shift settings.

Previous studies have shown that individual pathologists follow different precision-recall trade-offs during MC assessment^10,14^. As both under- and overestimation of mitotic figures can directly influence the tumor grade, precision and recall are equally important for MC assessment, which has motivated us to use the F_1_ score for algorithm evaluation. In a collaborative setting between algorithm and pathologist, lower detection thresholds might be favorable as discarding false positive mitotic figure detections might be easier than detecting missed candidates on the WSI.

## Usage Notes

To facilitate the use, we provide a Python Jupyter notebook to download all data automatically. All code examples are based on OpenSlide^37^ for WSI processing, Fastai v1^33^ for network training, Hydra^38^ for model configuration, and Weights & Biases^39^ for experiment tracking.

## Data Availability

We provide the code that we used to run all baseline experiments and all data in our GitHub repository (https://github.com/DeepMicroscopy/MIDOGpp).
