# Author Correction: A comprehensive multi-domain dataset for mitotic figure detection

**DOI:** 10.1038/s41597-024-03548-x

**Published:** 2024-07-02

**Authors:** Marc Aubreville, Frauke Wilm, Nikolas Stathonikos, Katharina Breininger, Taryn A. Donovan, Samir Jabari, Mitko Veta, Jonathan Ganz, Jonas Ammeling, Paul J. van Diest, Robert Klopfleisch, Christof A. Bertram

**Affiliations:** 1 Technische Hochschule Ingolstadt, Ingolstadt, Germany; 2 Pattern Recognition Lab, Friedrich-Alexander-Universität Erlangen-Nürnberg, Erlangen, Germany; 3 Department Artificial Intelligence in Biomedical Engineering, Friedrich-Alexander-Universität Erlangen-Nürnberg, Erlangen, Germany; 4 Department of Pathology, University Medical Center Utrecht, Utrecht, The Netherlands; 5 Schwarzman Animal Medical Center, New York, USA; 6 Department of Neuropathology, Universitätsklinikum Erlangen, Friedrich-Alexander-Universität Erlangen-Nürnberg, Erlangen, Germany; 7 Medical Image Analysis Group, Eindhoven University of Technology, Eindhoven, the Netherlands; 8 Institute of Veterinary Pathology, Freie Universität Berlin, Berlin, Germany; 9 Institute of Pathology, University of Veterinary Medicine Vienna, Vienna, Austria

**Keywords:** Prognostic markers, Tumour biomarkers, Translational research, Cancer, Prognostic markers, Tumour biomarkers, Translational research, Cancer

Correction to: *Scientific Data* 10.1038/s41597-023-02327-4, published online 25 July 2023

A bug in the original code for calculating the average precision values, reported as mean and standard deviation in Tables 5 and 7, meant that these values were incorrect in the original work. They have been updated in the PDF and HTML versions of the article. Copies of the old and new tables are appended below to highlight the changes. The updated code is available at https://github.com/DeepMicroscopy/MIDOGpp alongside the previous version (Commits f6cd999 and 8b15a5a).

Table 5 – old

**Table:** 

	breast carcinoma (human)		lung carcinoma (canine)		lymphosarcoma (canine)		cutaneous mast cell tumor (canine)		neuroendocrine tumor (human)		soft tissue sarcoma (canine)		melanoma (human)	
	mean	std	mean	std	mean	std	mean	std	mean	std	mean	std	mean	std
breast carcinoma (human)	0.54	0.04	0.28	0.07	0.22	0.05	0.43	0.06	0.28	0.05	0.29	0.03	0.63	0.04
lung carcinoma (canine)	0.32	0.01	0.49	0.04	0.38	0.02	0.30	0.06	0.16	0.03	0.39	0.03	0.40	0.03
lymphosarcoma (canine)	0.24	0.03	0.26	0.04	0.48	0.04	0.27	0.05	0.04	0.03	0.17	0.03	0.18	0.06
cutaneous mast cell tumor (canine)	0.37	0.05	0.22	0.08	0.23	0.07	0.38	0.05	0.15	0.06	0.25	0.04	0.36	0.15
neuroendocrine tumor (human)	0.29	0.02	0.17	0.05	0.22	0.04	0.32	0.09	0.30	0.02	0.21	0.07	0.59	0.05
soft tissue sarcoma (canine)	0.41	0.08	0.40	0.07	0.39	0.05	0.37	0.10	0.24	0.04	0.45	0.04	0.48	0.08
melanoma (human)	0.42	0.05	0.26	0.08	0.14	0.05	0.42	0.07	0.31	0.02	0.30	0.04	0.67	0.03
all	0.58	0.02	0.49	0.04	0.51	0.05	0.41	0.04	0.28	0.04	0.42	0.03	0.66	0.02

Table 5 – new

**Table:** 

	breast carcinoma (human)		lung carcinoma (canine)		lymphosarcoma (canine)		cutaneous mast cell tumor (canine)		neuroendocrine tumor (human)		soft tissue sarcoma (canine)		melanoma (human)	
	mean	std	mean	std	mean	std	mean	std	mean	std	mean	std	mean	std
breast carcinoma (human)	0.70	0.03	0.31	0.09	0.22	0.06	0.64	0.09	0.53	0.05	0.43	0.09	0.75	0.05
lung carcinoma (canine)	0.45	0.02	0.62	0.02	0.46	0.06	0.31	0.06	0.53	0.05	0.59	0.03	0.77	0.01
lymphosarcoma (canine)	0.44	0.05	0.50	0.05	0.80	0.01	0.67	0.03	0.55	0.01	0.48	0.05	0.59	0.05
cutaneous mast cell tumor (canine)	0.45	0.02	0.23	0.09	0.23	0.08	0.87	0.02	0.50	0.06	0.40	0.06	0.70	0.01
neuroendocrine tumor (human)	0.40	0.08	0.34	0.07	0.28	0.10	0.32	0.11	0.52	0.03	0.40	0.02	0.67	0.03
soft tissue sarcoma (canine)	0.55	0.05	0.48	0.05	0.41	0.05	0.54	0.11	0.38	0.09	0.69	0.04	0.60	0.09
melanoma (human)	0.46	0.09	0.30	0.10	0.14	0.05	0.46	0.11	0.55	0.03	0.52	0.02	0.80	0.01
all	0.74	0.02	0.65	0.06	0.69	0.04	0.82	0.01	0.62	0.03	0.69	0.03	0.82	0.03

Table 7 – old

**Table:** 

	breast carcinoma (human)		lung carcinoma (canine)		lymphosarcoma (canine)		cutaneous mast cell tumor (canine)		neuroendocrine tumor (human)		soft tissue sarcoma (canine)		melanoma (human)	
	mean	std	mean	std	mean	std	mean	std	mean	std	mean	std	mean	std
w/o breast carcinoma (human)	0.50	0.02	0.52	0.04	0.48	0.02	0.38	0.03	0.28	0.03	0.42	0.05	0.62	0.03
w/o lung carcinoma (canine)	0.52	0.03	0.37	0.08	0.45	0.07	0.38	0.08	0.27	0.05	0.37	0.09	0.63	0.06
w/o lymphosarcoma (canine)	0.55	0.05	0.44	0.03	0.41	0.01	0.46	0.09	0.31	0.05	0.42	0.03	0.65	0.04
w/o cutaneous mast cell tumor (canine)	0.57	0.01	0.50	0.01	0.51	0.01	0.50	0.04	0.30	0.03	0.44	0.03	0.67	0.03
w/o neuroendocrine tumor (human)	0.58	0.03	0.50	0.02	0.50	0.03	0.42	0.02	0.33	0.01	0.44	0.03	0.66	0.01
w/o soft tissue sarcoma (canine)	0.56	0.02	0.46	0.03	0.47	0.02	0.37	0.03	0.26	0.05	0.40	0.03	0.63	0.03
w/o melanoma (human)	0.55	0.04	0.43	0.07	0.43	0.05	0.37	0.04	0.23	0.05	0.38	0.03	0.61	0.04

Table 7 - new

**Table:** 

	breast carcinoma (human)		lung carcinoma (canine)		lymphosarcoma (canine)		cutaneous mast cell tumor (canine)		neuroendocrine tumor (human)		soft tissue sarcoma (canine)		melanoma (human)	
	mean	std	mean	std	mean	std	mean	std	mean	std	mean	std	mean	std
w/o breast carcinoma (human)	0.70	0.02	0.66	0.03	0.72	0.02	0.82	0.02	0.61	0.02	0.67	0.05	0.80	0.02
w/o lung carcinoma (canine)	0.71	0.05	0.65	0.05	0.67	0.08	0.81	0.05	0.61	0.04	0.70	0.02	0.79	0.04
w/o lymphosarcoma (canine)	0.71	0.04	0.64	0.03	0.71	0.03	0.81	0.04	0.60	0.02	0.68	0.02	0.80	0.02
w/o cutaneous mast cell tumor (canine)	0.72	0.02	0.64	0.03	0.65	0.12	0.79	0.06	0.62	0.01	0.69	0.01	0.82	0.02
w/o neuroendocrine tumor (human)	0.73	0.03	0.67	0.02	0.73	0.02	0.83	0.01	0.63	0.02	0.69	0.03	0.81	0.01
w/o soft tissue sarcoma (canine)	0.71	0.01	0.63	0.04	0.70	0.04	0.79	0.06	0.61	0.03	0.68	0.02	0.78	0.02
w/o melanoma (human)	0.71	0.04	0.59	0.08	0.60	0.16	0.77	0.05	0.60	0.03	0.67	0.04	0.79	0.03

